# Impact of diabetes on breast cancer mortality in elderly female patients: A retrospective analysis (1999–2020)

**DOI:** 10.1097/MD.0000000000048934

**Published:** 2026-05-22

**Authors:** Abu Huraira Bin Gulzar, Piyush Ratan, Ahmed Faizan, Khushbakht Waqar, Ashwa Riaz, Faraz Iqbal Khaskheli, Fareeda Brohi, Amir Hamza Khan, Sarah Zaka, Anza Shahid, Bismah Azam Ali, Misbah Bibi, Muhammad Ahtizaz Ahmed, Asad Ali Ahmed Cheema

**Affiliations:** a Department of Medicine, Services Institute of Medical Sciences, Lahore, Pakistan; b Department of Medicine, Patna Medical College, Patna, India; c Department of Medicine, Allama Iqbal Medical College, Lahore, Pakistan; d Faculty of Pharmacy & Pharmaceutical Sciences, University of Karachi, Karachi, Pakistan; e Department of Medicine, Shifa College of Medicine, Islamabad, Pakistan; f Department of Medicine, Liaquat University of Medical & Health Science, Jamshoro, Pakistan; g Department of Medicine, Peoples University of Medical and Health Sciences for Women, Nawabshah, Pakistan; h Department of Medicine, Rawalpindi Medical University, Rawalpindi, Pakistan; i Department of Medicine, Dow University of Health Sciences, Karachi, Pakistan; j Department of Medicine, Bahria University Health Sciences Campus, Karachi, Pakistan; k Department of Medicine, Henry Ford Macomb Hospital, Michigan; l Department of Medicine, International School of Medicine, International University of Kyrgyzstan, Bishkek, Kyrgyzstan.

**Keywords:** breast cancer, diabetes, disparities, mortality, trends

## Abstract

Breast cancer remains a leading cause of morbidity and mortality among women in the United States. Diabetes mellitus (DM) is also highly prevalent in the elderly female population, and the 2 conditions frequently coexist. National mortality trends in older women with both breast cancer and DM have not been thoroughly explored. We conducted a retrospective analysis of mortality data for women aged ≥65 years with breast cancer and DM using the CDC WONDER database (1999–2020). ICD-10 codes (breast cancer: C50; DM: E10–E14) were used to obtain de-identified patient data. age-adjusted mortality rates and annual percent changes were calculated using Joinpoint regression. Trends were analyzed to identify disparities among age, race/ethnicity, region, and urbanization level. A total of 53,327 deaths occurred in elderly female patients with breast cancer and DM in the United States between 1999 and 2020. The average annual percentage change of 0.3% (95% confidence interval: −0.4–0.9; *P* = .45). Mortality was highest among women aged >65 years (age-adjusted mortality rate [AAMR]: 20.59). Non-Hispanic (NH) Black women had the highest mortality (AAMR: 16.0), and regionally, the AAMR was highest in the Midwest (AAMR: 11.3). The states with the highest AAMR were the District of Columbia, Ohio, Nebraska, Oklahoma, and West Virginia. Additionally, nonmetropolitan areas had consistently higher AAMR (11.80) compared to metropolitan areas (9.40). Our study demonstrated a stable trend in the DM-related breast cancer mortality in recent years. Several high-risk groups warrant further investigations, including older elderly women (aged > 65), NH black females, the Midwestern population, nonmetro regions, and states in the top 10th percentile (District of Columbia, Ohio, Nebraska, Oklahoma, and West Virginia). Robust targeted policies and healthcare strategies are urgently needed to address disparities and reduce mortality in vulnerable populations.

## 1. Introduction

Breast cancer is the most frequently diagnosed malignancy in women in the United States. According to the SEER estimates, there will be 316,950 new cases in 2025, accounting for 15.5% of all cancer cases, while representing 30% of all female cancers across the US.^[1–3]^ Based on the data from 2019 to 2023, the incidence rate of breast cancer is around 130 cases per 100,000 women annually, and the lifetime risk of breast cancer is estimated to be one in 8.^[1]^ Although the relative survival rate of the disease is relatively favorable, at approximately 90 percent over a five-year period, it has a high prevalence rate, making it a significant burden to public health.^[4]^ In the United States, as of 2022, it is estimated that 4 million women have a history of breast cancer.^[1]^ This high prevalence, coupled with the increasing population of the elderly, highlights the necessity to explore pathological and metabolic comorbidities, especially diabetes mellitus (DM), which can further affect the outcomes of breast cancer. A meta-analysis has shown that women with diabetes have a significantly higher (~20%) risk of developing breast cancer compared to those without diabetes.^[5,6]^ Additionally, type 2 diabetes mellitus (T2DM) and hyperinsulinemia have been independently associated with an increased risk of postmenopausal breast cancer.^[5,7]^ T2DM and breast cancer share common risk factors, including obesity, family history, and alcohol use. T2DM may promote breast cancer development and progression through hyperinsulinemia, chronic hyperglycemia, and hormonal dysregulation. Breast cancer patients with diabetes exhibit poorer overall and disease-specific survival outcomes.

Breast cancer affects women of all ages; however, its incidence rises significantly with age, peaking between 45 and 64 years.^[8]^ There is increasing recognition that T2DM and breast cancer frequently coexist in the same patient population, contributing to higher mortality rates.^[8,9]^ Nearly 50% of women are 65 years or older at the time of breast cancer diagnosis, and prognosis is generally poorer in patients with both diabetes and breast cancer.^[5,10]^ According to a meta-analysis by Zhao XB et al, preexisting diabetes significantly worsens prognosis in breast cancer patients, with a 51% higher risk of death for overall survival (HR 1.51) and a 28% higher risk of poorer disease-free survival (HR 1.28), while no significant difference was observed for relapse-free period (HR 1.42).^[11]^

Investigating the link between diabetes and breast cancer-related mortality is imperative, given the increasing prevalence of both conditions and the limited comprehensive research on this topic. A detailed, country-specific epidemiological analysis of these co-morbidities is necessary for policymakers to evaluate the effectiveness of existing interventions, optimize resource allocation, and monitor progress at the national level.^[12]^ In addition, it is critical to identify these trends for refining risk assessment approaches, informing urgent care needs, developing prevention strategies, and promoting lifestyle modifications.^[13]^ Particular attention should be given to disparities in health outcomes based on socioeconomic status, race, gender, age, and geographic location.^[14]^ In the United States, similar evaluations are imperative and, to some extent, already underway through initiatives led by the Centers for Disease Control and Prevention (CDC), such as the Behavioral Risk Factor Surveillance System and the National Health And Nutrition Examination Survey, which aim to track chronic disease patterns and associated risk factors across diverse population subgroups.

In order to evaluate the mortality burden and to check the temporal trends in mortality, this study aims to identify potential demographic and racial disparities in breast cancer-related mortalities from 1999 to 2020 among elderly females within the United States, reflecting potential differences in healthcare access, socioeconomic factors, and treatment availability.

## 2. Methods

### 2.1. Study design and population setting

In this retrospective study, we sourced mortality data from the Centers for Disease Control and Prevention Wide-Ranging Online Data for Epidemiologic Research (CDC WONDER) database. We utilized death certificate data from 1999 to 2020 for breast cancer-related mortality in elderly females (65 years and above) with T2DM.^[15]^ Breast cancer patients were identified with International Classification of Diseases, 10^th^ Revision Clinical Modification (ICD-10-CM) code C50, and T2DM patients were identified with ICD-10-CM codes E10 to E14. The same ICD codes and age distribution have been used in previous studies.^[16–18]^ The Multiple Cause-of-Death Public Use records were analyzed to identify death records with both Breast Cancer and T2DM, mentioned as contributing causes of death on nationwide death certificates.

### 2.2. Ethical review

This study was exempt from the Institutional Review Board as we utilized mortality data from a de-identified publicly available database provided by the government, conforming to the Strengthening the Reporting of Observational Studies in Epidemiology guidelines.^[19]^

### 2.3. Data abstraction

Mortality data on elderly females with breast cancer and DM were abstracted between January 1999 and December 2020. State, year, region, race/ethnicity, urban-rural classification, and place of death were among the data gathered. Race/ethnicity categories included non-Hispanic (NH) White, NH Black or African American, Hispanic or Latino, and NH Asian or Pacific Islander. Based on the 2013 US Census, the population was classified into metropolitan and non-metropolitan areas as per the National Center for Health Statistics Urban-Rural scheme.^[20]^ The death locations included: Decedent’s home, Nursing home/long term care, Medical Facility (Inpatient), Other, Hospice facility, Medical Facility (Outpatient or ER). Census Regions were divided into Northeast, Midwest, South, and West categories using the U.S Census Bureau definitions.^[21]^ In order to prevent any bias in the analyses, deaths reported below 10 were deemed suppressed and were not used in the analysis. Moreover, data reported under the “unknown” category in demographic variables (e.g., age, race) were excluded from the analysis and not included in the totals to prevent bias in the results.

### 2.4. Statistical analysis

Using the 2000 US population as a baseline, we determined both crude and age-adjusted mortality rates (AAMR) per 100,000 population from 1999 to 2020.^[22]^ Deriving a 95% confidence interval (CI) from the database, the AAMRs were computed considering year, race/ethnicity, state, region, urban-rural classification, and place of death into account. To evaluate DM related mortality in elderly females with Breast Cancer, the Join Point Regression Program (version 5.3.0; National Cancer Institute, Montgomery County) was used to calculate annual percentage change (APC) with 95% CI in AAMR. By utilizing log-linear regression models, we assessed temporal trends in AAMR over time. Considering the 2-tailed *t*-testing, annual percent changes were found to be increasing or decreasing if the slope illustrating the change in mortality deviated significantly from zero. *P* value was considered statistically significant at <.05.

## 3. Results

A total of 53,327 deaths occurred in female breast cancer patients with diabetes aged ≥ 65 years in the United States between 1999 and 2020. Of which, 31.3% occurred in the decendant’s home, 30.4% in nursing homes or long-term care centers, 29% in medical facilities (inpatient; 24.2%, outpatient/ER; 4.3%, dead on arrival; 0.4%, and status unknown; 0.1%), 4.7% in other places, 4.4% in hospices, and 0.2% deaths had their place of death unknown (Table 1, Fig. S1, Table S1).

**Table 1:** Annual percent changes (APCs) and average annual percentage changes (AAPCs) of breast cancer and diabetes-related age-adjusted mortality rates per 100,000 in older females in the United States, 1999–2020.

Cohort	Segment	Lower endpoint	Upper endpoint	APCs (95% CI)	AAPCs (95% CI)	*P*-value
Overall						
Overall	1	1999	2006	0.41 (−0.45–1.27)	0.26 (−0.42–0.94)	.4508
	2	2006	2018	−1.84 (−2.3– −1.37)*		
	3	2018	2020	13.25 (6.01–20.98)*		
Age groups						
65–74 yr	1	1999	2018	−1.66 (−2.03–−1.29)*	−0.63 (−1.63–0.37)	.2165
	2	2018	2020	9.66 (−1.59–22.21)		
75–84 yr	1	1999	2006	0.85 (−0.38–2.10)	0.41 (−0.48–1.30)	.3708
	2	2006	2018	−1.94 (−2.56–−1.31)*		
	3	2018	2020	13.90 (4.68–23.92)*		
85+ yr	1	1999	2010	0.52 (−0.17–1.22)	0.67 (−0.16–1.50)	.1121
	2	2010	2017	−2.69 (−4.24–−1.11)*		
	3	2017	2020	9.53 (4.84–14.42)*		
Race						
Hispanic or Latino	1	1999	2018	−0.95 (−1.76–−0.12)*	0.51 (−1.27–2.32)	.5783
	2	2018	2020	15.42 (−4.14–38.98)		
Asian or Pacific Islander	1	1999	2020	0.04 (−0.9–0.99)	0.04 (−0.9–0.99)	.929
Black or African American	1	1999	2010	−0.16 (−1.2–0.9)	0.19 (−1.21–1.6)	.7946
	2	2010	2018	−3.4 (−5.25–−1.51)*		
	3	2018	2020	18.13 (3.81–34.43)*		
White	1	1999	2006	0.47 (−0.55–1.49)	0.1 (−0.57–0.78)	.7639
	2	2006	2017	−2.01 (−2.63–−1.38)*		
	3	2017	2020	7.34 (3.17–11.69)*		
Census regions						
Northeast	1	1999	2018	−2.05 (−2.47–−1.62)*	−0.7 (−1.94–0.55)	.2714
	2	2018	2020	13.01 (−1.33–29.44)		
Midwest	1	1999	2008	0.24 (−0.61–1.11)	−0.49 (−1.43–0.45)	.3061
	2	2008	2018	−3.47 (−4.34–−2.6)*		
	3	2018	2020	12.06 (2.28–22.76)*		
South	1	1999	2001	6.43 (−4.02–18.02)	1.5 (0.28–2.74)	.0155
	2	2001	2018	−0.72 (−1.13–−0.31)*		
	3	2018	2020	16.9 (7.28–27.38)*		
West	1	1999	2020	−0.24 (−0.71–0.24)	−0.24 (−0.71–0.24)	.3115
Urbanization						
Metropolitan	1	1999	2002	2.87 (−1.48–7.42)	0.35 (−0.64–1.35)	.4904
	2	2002	2018	−1.46 (−1.84–−1.07)*		
	3	2018	2020	11.8 (2.31–22.17)*		
Non Metropolitan	1	1999	2005	1.49 (−0.24–3.25)	0.58 (−0.41–1.58)	.2504
	2	2005	2018	−1.69 (−2.28–−1.09)*		
	3	2018	2020	13.55 (3.49–24.59)*		

CI = confidence interval.

* The APC is significantly different from zero at α = 0.05.

### 3.1. Annual trends for breast cancer and diabetes-related AAMR

**Figure 1.**
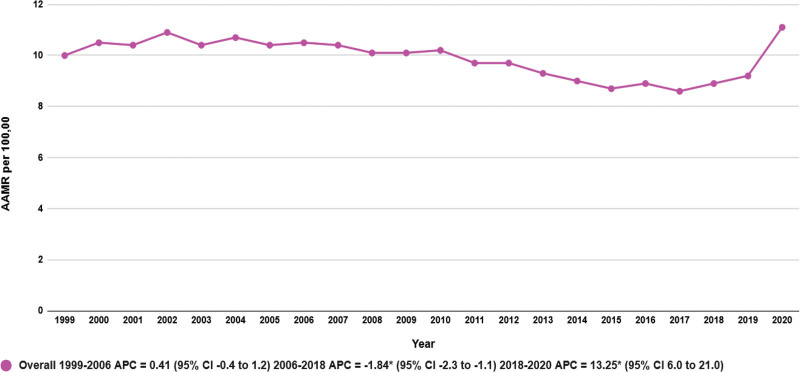
Diabetes-related breast cancer AAMRs per 100,000 adults in the United States from 1999 to 2020. *The APC is significantly different from zero at α = 0.05. AAMR = age-adjusted mortality rate, APC = annual percent change, CI = confidence intervals.

The AAMR for breast cancer and DM related deaths increased from 10.0 in 1999 to 11.10 in 2020. The overall AAMR remained stable from 1999 to 2006 (APC: 0.41; 95% CI: −0.45–1.27, *P* = .32), followed by a steady decrease from 2006 to 2018 (APC: −1.84; 95% CI: −2.30–−1.37, *P* < .001). Finally, there was a moderate rise in the AAMR from 2018 to 2020 (APC: 13.25; 95% CI: 6.0–21.0, *P* = .001) (Table 1, Fig. 1, Table S2).

### 3.2. Breast cancer and diabetes-related mortality stratified by demographics

#### 3.2.1. Age group analysis

The 85+ age group demonstrated the highest mortality (overall CMR: 20.59; 95% CI: 20.28–20.91), followed by the 75 to 84 years age group (CMR: 12.16; 95% CI: 11.99–12.32) and the 65 to 74 years age group (CMR: 5.74; 95% CI: 5.65–5.83).

**Figure 2.**
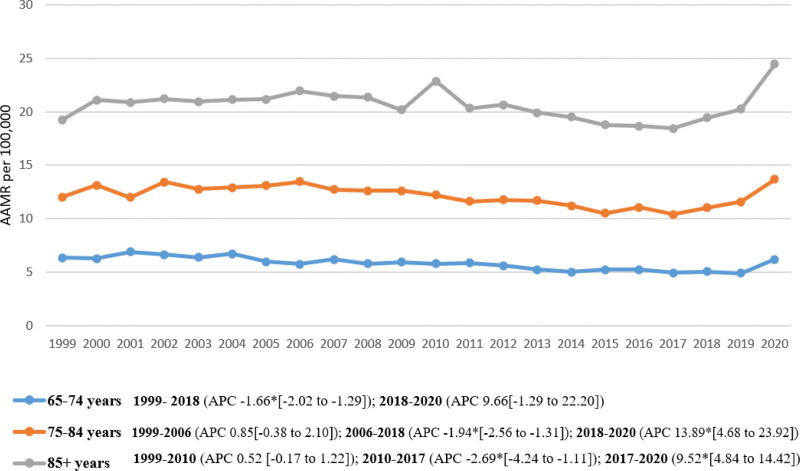
Diabetes-related breast cancer AAMRs per 100,000 adults in the United States from 1999 to 2020 stratified by age group. *The APC is significantly different from zero at α = 0.05. AAMR = age-adjusted mortality rate, APC = annual percent change, CI = confidence intervals.

Mortality for the 85+ age group remained stable from 1999 to 2010 (APC: 0.52; 95% CI: −0.17–1.22), followed by a decrease between 2010 and 2017 (APC: −2.69; 95% CI: −4.24–−1.11) and an increase until 2020 (APC: 9.52; 95% CI: 4.84–14.42). The 75 to 84 years age group showed a constant mortality rate from 1999 to 2006 (APC: 0.85; 95% CI: −0.38–2.10), followed by a decrease until 2018 (APC: −1.94; 95% CI: −2.56–−1.31) and an increase until 2020 (APC: 13.89; 95% CI: 4.68–23.92). The 65 to 74 years age group showed a declining trend between 1999 and 2018 (APC: −1.66; 95% CI: −2.02–−1.29) and an increase until 2020 (APC: 9.66; 95% CI: −1.29–22.20) (Table 1, Fig. 2, Table S3).

#### 3.2.2. Race

When stratified by race/ethnicity, AAMRs were highest among NH Blacks or African Americans, followed by NH Whites, Hispanics or Latinos, and NH Asian or Pacific Islanders (overall AAMR NH Blacks: 16.0; 95% CI: 15.70–16.40; NH Whites: 9.30; 95% CI: 9.20–9.40; Hispanics: 8.70; 95% CI: 8.40–9.10; NH Asians: 6.0; 95% CI: 5.70–6.40).

**Figure 3.**
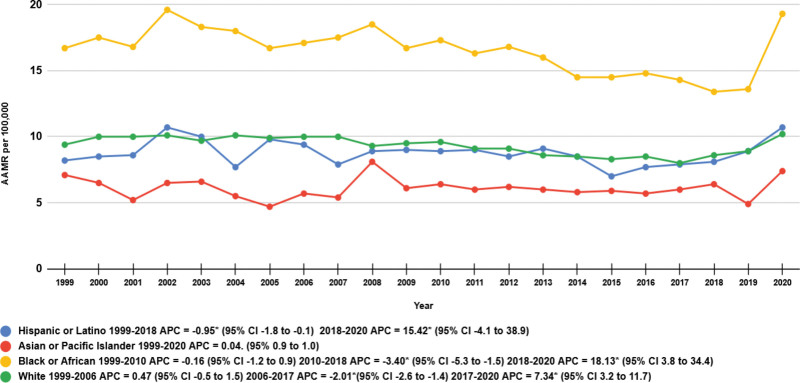
Diabetes-related breast cancer AAMRs per 100,000 adults in the United States from 1999 to 2020 stratified by Race. *The APC is significantly different from zero at α = 0.05. AAMR = age-adjusted mortality rate, APC = annual percent change, CI = confidence intervals.

Joinpoint regression analysis revealed that the AAMRs for NH Blacks remained steady from 1999 to 2010 (APC: −0.16; 95% CI: −1.20–0.90, *P* = .75). This was followed by a subsequent decrease from 2010 to 2018 (APC: −3.40; 95% CI: −5.25–−1.51, *P* = .001), and finally an uptick from 2018 to 2020 (APC: 18.13; 95% CI: 3.80–34.43, *P* = .01). AAMRs for NH Whites showed a more uniform distribution, with mortality remaining more or less constant from 1999 to 2006 (APC: 0.47; 95% CI: −0.55–1.49, *P* = .34), then a slight dip from 2006 to 2017 (APC: −2.01; 95% CI: −2.63–−1.38, *P* < .001), and finally an increase from 2017 to 2020 (APC: 7.34; 95% CI: 3.17–11.69, *P* = .001). AAMRs for Hispanics showed little variation from 1999 to 2018 (APC: −0.95; 95% CI: −1.76–−0.12, *P* = .02) followed by a rise from 2018 to 2020 (APC: 15.42; 95% CI: −4.14–38.97, *P* = .12). Mortality rates for Asians remained stable throughout the study period of 1999 to 2020 (APC: 0.04; 95% CI: 0.90–0.98, *P* = .93) (Table 1, Fig. 3, Table S4).

#### 3.2.3. Census region and state

Throughout the study period, the highest mortality was observed in the Midwestern region (overall AAMR: 11.30; 95% CI: 11.10–11.50), followed by the Western (AAMR: 9.60; 95% CI: 9.40–9.80), Northeastern (AAMR: 9.50; 95% CI: 9.30–9.70) and Southern (AAMR: 9.20; 95% CI: 9.10–9.40) regions.

The AAMRs for every region closely approximated each other throughout the study period. AAMRs for the Northeast remained stable from 1999 to 2007 (APC: −0.72; 95% CI: −2.22–0.80), dipped from 2007 to 2017 (APC: −3.21; 95% CI: −4.47–−1.92) and increased until 2020 (APC: 9.56; 95% CI: 2.71–16.87). The Midwest showed a similar trend (1999–2008 АРС: 0.24; 95% CI: −0.60–1.10, *P* = .55; 2008–2018 АРС: −3.47; 95% CI: −4.33–2.60, *P* < .001; 2018–2020 АРС: 12.06; 95% CI: 2.28–22.76, *P* = .01). The South showed a steady increase in mortality (1999–2001 АРС: 6.43; 95% CI: −4.02–18.02, *P* = .21; 2001–2018 APC: −0.72; 95% CI: −1.13–−0.31, *P* = .002; 2018–2020 АРС: 16.90; 95% CI: 7.28–27.38, *P* = .001), while the West had no major fluctuations (1999–2020 APC: −0.24; 95% CI: −0.71–0.24, *P* = .31) (Table 1, Fig. S2, Table S5).

**Figure 4.**
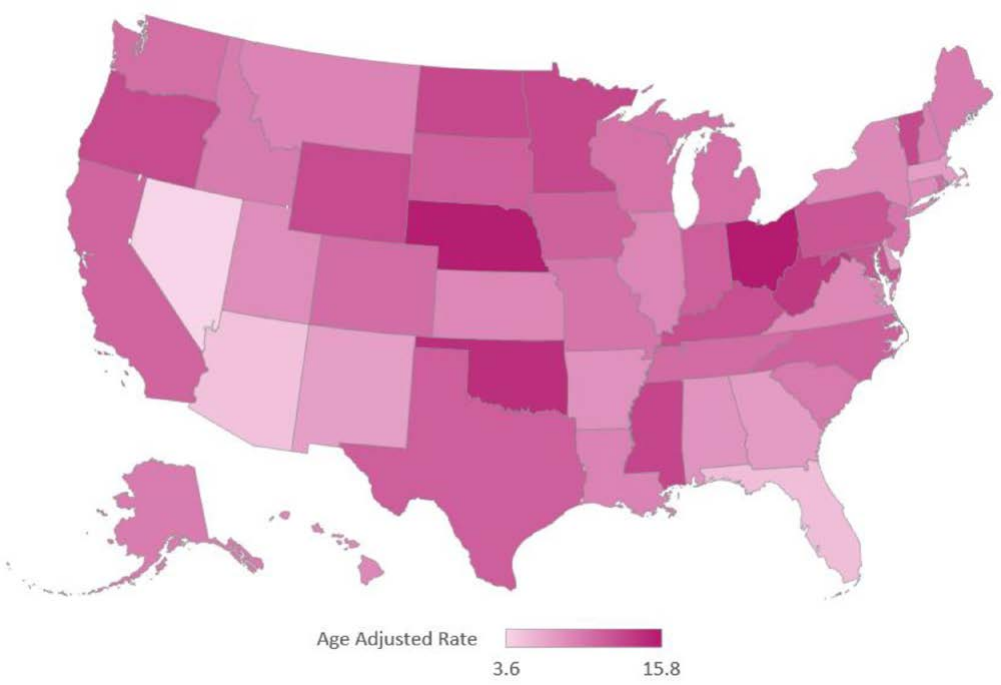
Diabetes-related breast cancer AAMRs per 100,000 adults in the United States from 1999 to 2020 stratified by State. AAMR = age-adjusted mortality rate.

There was a large difference in AAMRs between different states, ranging from 3.6 (95% CI: 3.0–4.2) in Nevada to 15.8 (95% CI: 13.30–18.30) in the District of Columbia. States in the top 90^th^ percentile (District of Columbia, Ohio, Nebraska, Oklahoma, and West Virginia) had approximately more than 4 times the AAMRs as compared to those in the lower 10^th^ percentile (Massachusetts, New Mexico, Florida, Arizona, and Nevada) (Fig. 4, Table S6).

#### 3.2.4. Urbanization

Nonmetropolitan areas had higher overall AAMR (11.80; 95% CI: 11.60–12.0) as compared to metropolitan areas (9.40; 95% CI: 9.30–9.50) throughout the study period. Metropolitan areas first experienced a rise in mortality from 1999 to 2002 (APC: 2.87; 95% CI: −1.48–7.42, *P* = .18), followed by a decrease until 2018 (APC: −1.45; 95% CI: −1.84–−1.07, *P* < .001) and finally a steady increase until 2020 (APC: 11.80; 95% CI: 2.31–22.17, *P* = .01). Nonmetropolitan areas showed a very similar trend, with mortality increasing gradually from 1999 to 2005 (APC: 1.49; 95% CI: −0.24–3.25, *P* = .08), declining slightly until 2018 (APC: −1.69; 95% CI: −2.28–−1.09, *P* = .00003), and culminating with an increase until 2020 (APC: 13.55; 95% CI: 3.48–24.60, *P* = .01) (Table 1, Fig. 3, Table S7).

## 4. Discussion

This study offers a detailed examination of breast cancer mortality trends among older women with diabetes in the United States from 1999 to 2020, addressing critical gaps in the understanding of how diabetes influences breast cancer.

It was noted that the AAMRs of the women aged 85+ years were consistently higher than those between aged 65 to 74 years and 75 to 84 years. Moreover, the populations with the highest AAMR were those who identified as NH Black or African American; these groups were followed by NH whites, Hispanics, and NH Asian and Pacific Islanders. Based on regional disparities, the AAMR was highest in the Midwest, followed by the Northeast, the West, and then the South. In general, AAMRs were higher in non-metropolitan areas than in metropolitan areas. Understanding these trends is crucial to drive targeted interventions and healthcare policy, which in turn will improve outcomes for the senior population and lessen the burden of diabetes associated breast cancer deaths.

Our findings reinforce the established association between breast cancer and diabetes.^[23,24]^ DM and breast cancer are 2 prevalent chronic diseases that significantly impact global mortality. Studies suggest a bidirectional relationship between diabetes and breast cancer. The link between DM and breast cancer has been hypothesized to be related to hormonal imbalance and chronic inflammation. Firstly, insulin resistance and hyperinsulinemia in diabetes activate IGF and insulin receptors, promoting cancer growth, progression, and chemoresistance through multiple pathways.^[25]^ Secondly, it is also proposed that hyperglycemia influences tumor cells by increasing proliferation, inducing mutations, augmenting invasion and migration, as well as altering cancer-related signaling pathways.^[26]^ Thirdly, poorly controlled diabetes creates a pro-inflammatory condition which promotes genetic instability and is associated with increased cancer risk.^[27]^ Therefore, insulin resistance, hyperglycemia, and poor diabetic control can potentially increase the risk of breast cancer and, over time, worsen the prognosis in women with already diagnosed breast cancer. A study by Luo J et al 2015 strengthens the association of diabetes in worsening the prognosis of breast cancer in women. In their cohort of 3000 women, they found that preexisting diabetes was linked to 57% higher overall mortality and 36% higher cancer-specific mortality.^[24]^

This study demonstrates a long-term stability in AAMRs across all the groups, with the highest rates observed in NH Black women. Racial/ethnic disparities in breast cancer are driven by factors such as lack of medical coverage, more advanced disease, and unequal access to cancer treatment.^[28]^ Historical racial and ethnic segregation, disparities in income, education, and other social determinants of health, may contribute to health inequities in the U.S.^[29]^ The dramatic rise in AAMR from 2018 to 2020 marks a deterioration in the health of individuals, which could be attributed to multiple factors, with COVID-19 being the foremost. Moreover, the African American community was disproportionately affected by the combination of poor social policies, programs, biased economic arrangements, and politics.^[30]^ The COVID-19 pandemic resulted in an economic recession, and subsequently, an estimated 7.3 million people lost employer-sponsored insurance coverage.^[31]^ Thus, the pandemic era, followed by the unequal availability of resources and the overburdened healthcare system, could have potentially contributed to the increase in AAMRs in all groups, especially affecting the minorities. In contrast, the NH White population demonstrated a rather consistent pattern of mortality over time before increasing significantly in 2017. The initial dip from 2006 to 2017 can be explained by public health efforts to address healthcare-related concerns such as early detection, screening, and treatment. From 2006 through 2015, breast cancer death rates declined annually by 2.6% in AI/ANs, 1.8% in NHWs, 1.5% in NHBs, 1.4% in Hispanics, and 0.9% in APIs.^[32]^ The subsequent rise from 2017 to 2020 could be potentially attributed to several factors, including more advanced cancer diagnoses in older women, reduced use of aggressive treatment, including adjuvant hormone therapy or chemotherapy,^[33]^ and significant disruptions to cancer care with the advent of the COVID-19 pandemic.^[34]^ The Hispanic population exhibited lower AAMRs compared to their NH counterparts. This could be attributed to limited access to healthcare due to language barriers or immigration status. Semi-structured qualitative interviews with Latinx caregivers demonstrated many concerning trends: caregivers commonly experienced emotional stress when communicating with clinicians, and patients frequently experienced communication without professional interpretation.^[35]^ The dissatisfaction and mistrust created by a patient’s immigration status impacted disease management. Finally, undocumented immigrants are often targeted for deportation, and the anxiety created by the fear of deportation impacts their mental health.^[36]^ The AAMRs for NH Asians and Pacific Islanders remained stable from 1999 to 2020. This could possibly be explained by the heterogeneity within this group, which may lead to varying health needs and better healthcare outcomes. Immigrants tend to be healthier than their nonimmigrant counterparts in the United States – a trend often referred to as the immigrant health advantage – may also contribute to this.^[37]^ Additionally, compared to Whites with cancer, Asian Americans with cancer had similar or better levels of access to care. Despite this, our study findings have been contradicted by a few studies in the past, which emphasized the need to further explore the unique health challenges faced by Asian American populations, access to healthcare, and underreporting of health disparities.

In addition, we also observed significant geographical variations in breast cancer and diabetes related mortality amongst elderly women, with the Midwest having the highest burden compared to the other regions of the United States.

Compared to the other census regions, the Midwest saw a relatively slower drop in mortality rates, an average annual decrease of 1.7% as opposed to the 2.1% yearly decline observed in the Northeast during the same period.^[38]^ Disparity in healthcare access and socioeconomic variables that impede early detection and optimal treatment might be the cause of the slower drop seen in the Midwest.^[39]^ Even though the elderly population is most at risk of acquiring cancer, regional disparities in geographic access to care, as measured by the number of oncologists per capita, do not correspond with regions with a greater elderly population.^[40]^ In the Midwest, diabetes related mortality also shows concerning trends, particularly in rural communities. A study indicated that U.S. rural counties have greater rates of diabetes related mortality than metropolitan areas, with the Midwest and Southern regions being specifically affected.^[41]^ Similarly, several U.S. states, particularly in the Midwest and South, exhibit significantly higher AAMRs for diabetes related breast cancer. Notably, Ohio, Nebraska, Mississippi, West Virginia, and Oklahoma consistently rank among the highest. As described above, these elevated mortality rates are frequently associated with racial disparities, limitations to healthcare access, higher prevalence of comorbid conditions such as obesity and hypertension, socioeconomic difficulties, and limited healthcare access.^[39,42]^ The high AAMR frequency in these regions highlights the need for geographically targeted interventions to improve early detection, chronic disease management, and accessibility to equitable preventive care services.

Another important factor is educational attainment; women with less education are less likely to follow preventive guidelines, such as getting mammograms on time. Poorer results and late-stage diagnosis are caused by such noncompliance.^[43]^ Targeted public health initiatives that enhance healthcare accessibility in marginalized and rural areas are necessary to address these discrepancies. Reducing diabetes related breast cancer mortality in the Midwest requires extending preventative screening programs, improving access to primary health care services, and bringing community-based health education programs into practice. Collaborative efforts involving policymakers, healthcare providers, and community organizations are crucial to develop and implement strategies that address the individual challenges faced by these populations.

Our findings substantiate the need for public health considerations. It is necessary to consider the impact of race,^[28]^ socioeconomic status, access to care, geographical location, and other factors on the detection, treatment, and prognosis of breast cancer in elderly women with diabetes. Gaps like limited access to healthcare, along with socioeconomic challenges,^[44]^ lead to worse breast cancer-related outcomes; thus, we need more nuanced, community-specific approaches to improve outcomes. Particularly, diabetic women are often diagnosed with breast cancer at a later stage, in part because they are less likely to undergo regular screening. To promote earlier diagnoses, screening tests such as mammography and breast MRI should be promoted.^[45]^ Lastly, preventive measures like lifestyle modifications should be promoted through public health campaigns, for example, encouraging routine physical activity and a healthy diet, which can reduce breast cancer risk while also supporting diabetes management; thus, diabetes-oncology integrated management programs can be potentially effective.^[46–50]^

Several limitations should be considered when interpreting the observed disparities in diabetes associated breast cancer mortality. First, because our analysis relies on death certificates and ICD coding, there is potential for misclassification or underreporting of breast cancer as the underlying cause of death. Such inaccuracies may differ by geographic region or socioeconomic context, potentially biasing comparisons of age- and race-specific mortality rates. Second, the CDC database lacks detailed clinical information, for example estrogen and progesterone receptor (ER/PR) status, HER2/neu expression, tumor grade, size, lymph node involvement, Ki-67 proliferation index, molecular subtype, and TNM stage. Without these variables, it is difficult to determine whether differences in mortality across racial or regional groups reflect variations in disease biology or disparities in diagnosis and treatment. Third, treatment-related data – including surgery, chemotherapy, radiotherapy, and targeted or hormonal therapies – are not available. This limitation restraints our ability to assess whether the higher mortality observed among NH Black women, older age groups, and residents of non-metropolitan areas may be attributed to differences in access to or quality of breast cancer care. Lastly, the database lacks socioeconomic indicators such as income, education, and insurance status, which are critical determinants of healthcare access and outcomes. The absence of these variables limits our ability to understand the impact of social disadvantage from biological or regional factors underlying the observed disparities.

Even with these limitations, our findings point to ongoing inequities that need to be addressed through focused interventions and better allocation of resources, so that older adults and underserved communities can have improved breast cancer outcomes.

## 5. Conclusion

Our study demonstrates that, between 1999 and 2020, breast cancer–and diabetes-related mortality among elderly females in the United States showed overall long-term stability with a notable late-period increase from 2018 to 2020. Several high-risk groups need to be studied in detail, such as older females (aged > 65), specifically NH Black females, the Midwestern population, nonmetropolitan regions, decedents’ homes as the place of death, and states in the top 10th percentile (District of Columbia, Ohio, Nebraska, Oklahoma, and West Virginia). Robust policies and strategic management systems are required to be developed and implemented to distribute the burden and effectively control this situation.

## Author contributions

**Conceptualization:** Abu Huraira Bin Gulzar, Piyush Ratan.

**Data curation:** Abu Huraira Bin Gulzar, Faraz Iqbal Khaskheli, Sarah Zaka.

**Methodology:** Anza Shahid.

**Project administration:** Anza Shahid.

**Resources:** Faraz Iqbal Khaskheli.

**Software:** Sarah Zaka, Anza Shahid.

**Supervision:** Abu Huraira Bin Gulzar.

**Visualization:** Amir Hamza Khan.

**Writing – original draft:** Piyush Ratan, Ahmed Faizan, Khushbakht Waqar, Ashwa Riaz, Amir Hamza Khan, Bismah Azam Ali, Misbah Bibi, Muhammad Ahtizaz Ahmed.

**Writing – review & editing:** Abu Huraira Bin Gulzar, Ahmed Faizan, Khushbakht Waqar, Ashwa Riaz, Faraz Iqbal Khaskheli, Fareeda Brohi, Bismah Azam Ali, Muhammad Ahtizaz Ahmed, Asad Ali Ahmed Cheema.

## Correction

This article was originally published with an incorrect affiliation for author “Khushbakht Waqar.” The correct affiliation has now been updated online from “*Department of Medicine, University of Karachi, Karachi, Pakistan*” to “*Faculty of Pharmacy & Pharmaceutical Sciences, University of Karachi, Karachi, Pakistan*.”




















