# Supplementary material for: Impact of diabetes on breast cancer mortality in elderly female patients: A retrospective analysis (1999–2020)
Source: Medicine (Baltimore). 2026 May 22;105(21):e48934. doi: 10.1097/MD.0000000000048934 (PMC13200986; doi:10.1097/MD.0000000000048934)
Supplement: Supplementary file 1 [file medi-105-e48934-s001.docx]

**
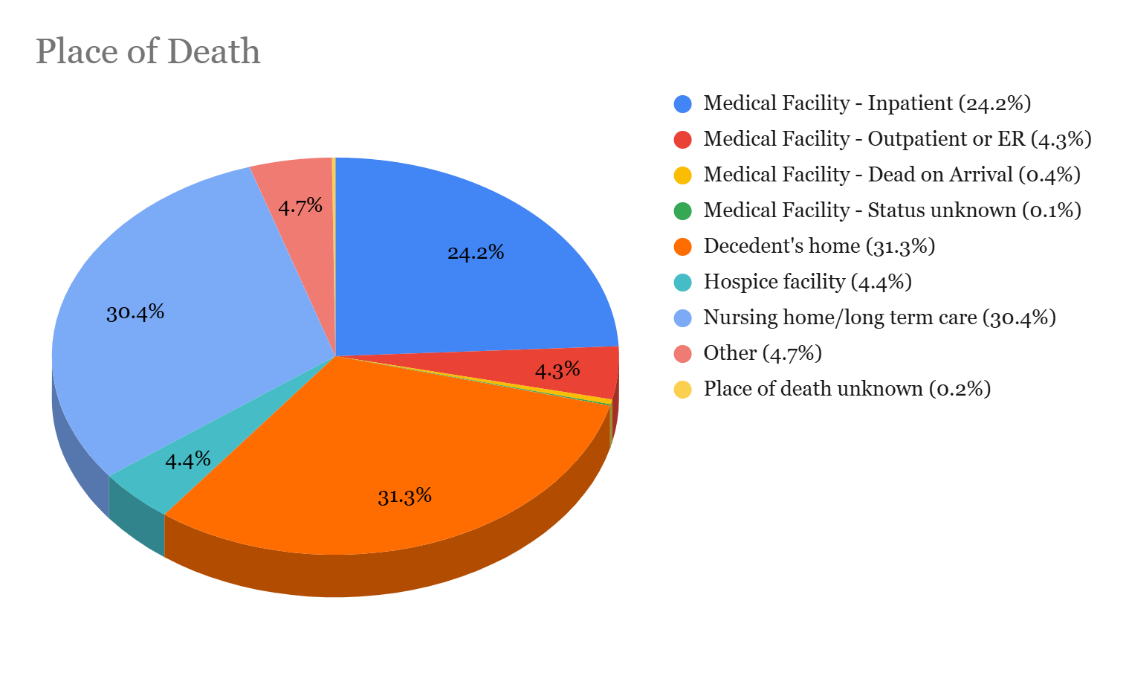
**

**Supplementary Figure 1.** Diabetes-related Breast Cancer mortality stratified by place of death in the United States from 1999 to 2020.
