# Supplementary material for: Impact of diabetes on breast cancer mortality in elderly female patients: A retrospective analysis (1999–2020)
Source: Medicine (Baltimore). 2026 May 22;105(21):e48934. doi: 10.1097/MD.0000000000048934 (PMC13200986; doi:10.1097/MD.0000000000048934)
Supplement: Supplementary file 2 [file medi-105-e48934-s002.docx]

| **Place of Death** | **Deaths** | **% of Total Deaths** |
| --- | --- | --- |
| Medical Facility - Inpatient | 12,931 | 24.2 |
| Medical Facility - Outpatient or ER | 2,270 | 4.3 |
| Medical Facility - Dead on Arrival | 198 | 0.4 |
| Medical Facility - Status unknown | 31 | 0.1 |
| Decedent's home | 16,711 | 31.3 |
| Hospice facility | 2,341 | 4.4 |
| Nursing home/long term care | 16,218 | 30.4 |
| Other | 2,497 | 4.7 |
| Place of death unknown | 130 | 0.2 |
| **Total (100%)** | **53,327** | **100** |

**Supplementary Table 1.**  Diabetes-related Breast Cancer mortality stratified by place of death in the United States from 1999 to 2020.
