# Supplementary material for: Impact of diabetes on breast cancer mortality in elderly female patients: A retrospective analysis (1999–2020)
Source: Medicine (Baltimore). 2026 May 22;105(21):e48934. doi: 10.1097/MD.0000000000048934 (PMC13200986; doi:10.1097/MD.0000000000048934)
Supplement: Supplementary file 3 [file medi-105-e48934-s003.docx]

| **Year** | **Overall/Female** |
| --- | --- |
| **1999** | 10(9.5-10.4) |
| **2000** | 10.5(10.1-11) |
| **2001** | 10.4(10-10.9) |
| **2002** | 10.9(10.4-11.3) |
| **2003** | 10.4(10-10.9) |
| **2004** | 10.7(10.2-11.1) |
| **2005** | 10.4(10-10.8) |
| **2006** | 10.5(10.1-10.9) |
| **2007** | 10.4(10-10.8) |
| **2008** | 10.1(9.7-10.5) |
| **2009** | 10.1(9.7-10.5) |
| **2010** | 10.2(9.8-10.6) |
| **2011** | 9.7(9.3-10.1) |
| **2012** | 9.7(9.3-10) |
| **2013** | 9.3(8.9-9.7) |
| **2014** | 9(8.6-9.3) |
| **2015** | 8.7(8.4-9.1) |
| **2016** | 8.9(8.6-9.3) |
| **2017** | 8.6(8.2-8.9) |
| **2018** | 8.9(8.6-9.3) |
| **2019** | 9.2(8.8-9.5) |
| **2020** | 11.1(10.7-11.5) |

**Supplementary Table 2.**  Diabetes-related Breast Cancer mortality among elderly females in the United States from 1999 to 2020.
