# Supplementary material for: Impact of diabetes on breast cancer mortality in elderly female patients: A retrospective analysis (1999–2020)
Source: Medicine (Baltimore). 2026 May 22;105(21):e48934. doi: 10.1097/MD.0000000000048934 (PMC13200986; doi:10.1097/MD.0000000000048934)
Supplement: Supplementary file 4 [file medi-105-e48934-s004.docx]

| **Year** | **65-74 years** | **75-84 years** | **85+ years** |
| --- | --- | --- | --- |
| **1999** | 6.35(5.86-6.84) | 12.01(11.22-12.8) | 19.25(17.67-20.83) |
| **2000** | 6.27(5.79-6.76) | 13.13(12.3-13.95) | 21.11(19.47-22.75) |
| **2001** | 6.9(6.39-7.41) | 12(11.22-12.78) | 20.88(19.25-22.5) |
| **2002** | 6.66(6.15-7.16) | 13.44(12.62-14.26) | 21.22(19.59-22.85) |
| **2003** | 6.38(5.89-6.87) | 12.78(11.99-13.58) | 20.94(19.34-22.54) |
| **2004** | 6.73(6.22-7.23) | 12.92(12.12-13.72) | 21.13(19.53-22.73) |
| **2005** | 5.99(5.52-6.47) | 13.09(12.28-13.9) | 21.17(19.59-22.75) |
| **2006** | 5.76(5.3-6.22) | 13.46(12.64-14.28) | 21.96(20.37-23.54) |
| **2007** | 6.2(5.73-6.67) | 12.71(11.91-13.51) | 21.47(19.93-23.02) |
| **2008** | 5.78(5.33-6.23) | 12.62(11.82-13.41) | 21.37(19.85-22.89) |
| **2009** | 5.95(5.5-6.4) | 12.61(11.81-13.41) | 20.18(18.72-21.64) |
| **2010** | 5.8(5.36-6.24) | 12.2(11.41-12.98) | 22.87(21.33-24.41) |
| **2011** | 5.86(5.42-6.29) | 11.63(10.86-12.39) | 20.32(18.9-21.75) |
| **2012** | 5.63(5.22-6.04) | 11.76(10.99-12.53) | 20.67(19.25-22.09) |
| **2013** | 5.23(4.84-5.62) | 11.71(10.94-12.47) | 19.93(18.55-21.31) |
| **2014** | 5.02(4.65-5.39) | 11.23(10.49-11.98) | 19.51(18.15-20.87) |
| **2015** | 5.23(4.86-5.6) | 10.52(9.8-11.23) | 18.79(17.47-20.12) |
| **2016** | 5.25(4.89-5.61) | 11.08(10.36-11.81) | 18.65(17.34-19.96) |
| **2017** | 4.96(4.61-5.31) | 10.42(9.73-11.12) | 18.45(17.15-19.75) |
| **2018** | 5.06(4.71-5.41) | 11.02(10.32-11.72) | 19.44(18.11-20.77) |
| **2019** | 4.92(4.58-5.25) | 11.59(10.89-12.3) | 20.24(18.89-21.6) |
| **2020** | 6.2(5.83-6.57) | 13.68(12.92-14.43) | 24.44(22.95-25.92) |

**Supplementary Table 3.** Diabetes-related Breast Cancer AAMR per 100,000 stratified by age group in the United States from 1999 to 2020.
