# Supplementary material for: Impact of diabetes on breast cancer mortality in elderly female patients: A retrospective analysis (1999–2020)
Source: Medicine (Baltimore). 2026 May 22;105(21):e48934. doi: 10.1097/MD.0000000000048934 (PMC13200986; doi:10.1097/MD.0000000000048934)
Supplement: Supplementary file 5 [file medi-105-e48934-s005.docx]

| **Age- Adjusted Rate (95% CI)** | | | | |
| --- | --- | --- | --- | --- |
| **Year** | **Hispanic or Latino** | **NH Asian or Pacific Islander** | **NH Black or African American** | **NH White** |
| **1999** | 8.2(6.4-10.4) | 7.1(4.7-10.2) | 16.7(14.8-18.6) | 9.4(8.9-9.8) |
| **2000** | 8.5(6.7-10.5) | 6.5(4.3-9.5) | 17.5(15.5-19.4) | 10(9.5-10.5) |
| **2001** | 8.6(6.9-10.7) | 5.2(3.4-7.8) | 16.8(14.9-18.7) | 10(9.5-10.5) |
| **2002** | 10.7(8.7-12.7) | 6.5(4.6-9.1) | 19.6(17.5-21.6) | 10.1(9.6-10.5) |
| **2003** | 10(8.1-11.9) | 6.6(4.6-9.2) | 18.3(16.3-20.3) | 9.7(9.3-10.2) |
| **2004** | 7.7(6.2-9.4) | 5.5(3.8-7.9) | 18(16-19.9) | 10.1(9.7-10.6) |
| **2005** | 9.8(8.1-11.6) | 4.7(3.1-6.8) | 16.7(14.9-18.6) | 9.9(9.5-10.4) |
| **2006** | 9.4(7.7-11.1) | 5.7(4-7.9) | 17.1(15.2-18.9) | 10(9.5-10.4) |
| **2007** | 7.9(6.4-9.4) | 5.4(3.8-7.5) | 17.5(15.7-19.4) | 10(9.5-10.4) |
| **2008** | 8.9(7.3-10.5) | 8.1(6.1-10.4) | 18.5(16.6-20.4) | 9.3(8.9-9.7) |
| **2009** | 9(7.5-10.5) | 6.1(4.5-8.1) | 16.7(14.9-18.5) | 9.5(9.1-10) |
| **2010** | 8.9(7.4-10.4) | 6.4(4.8-8.4) | 17.3(15.5-19.1) | 9.6(9.1-10) |
| **2011** | 9(7.6-10.5) | 6(4.5-7.9) | 16.3(14.6-18) | 9.1(8.7-9.6) |
| **2012** | 8.5(7.2-9.9) | 6.2(4.7-8.1) | 16.8(15-18.5) | 9.1(8.7-9.5) |
| **2013** | 9.1(7.7-10.4) | 6(4.6-7.8) | 16(14.4-17.7) | 8.6(8.2-9) |
| **2014** | 8.5(7.2-9.8) | 5.8(4.5-7.5) | 14.5(13-16.1) | 8.5(8.1-8.9) |
| **2015** | 7(5.8-8.1) | 5.9(4.5-7.5) | 14.5(13-16) | 8.3(7.9-8.7) |
| **2016** | 7.7(6.5-8.9) | 5.7(4.4-7.2) | 14.8(13.3-16.3) | 8.5(8.1-8.9) |
| **2017** | 7.9(6.8-9.1) | 6(4.8-7.5) | 14.3(12.8-15.7) | 8(7.6-8.3) |
| **2018** | 8.1(6.9-9.2) | 6.4(5.2-7.9) | 13.4(12.1-14.8) | 8.6(8.2-9) |
| **2019** | 8.9(7.7-10.1) | 4.9(3.8-6.2) | 13.6(12.3-15) | 8.9(8.5-9.2) |
| **2020** | 10.7(9.4-12) | 7.4(6-8.7) | 19.3(17.7-20.9) | 10.2(9.8-10.7) |

**Supplementary Table 4.** Diabetes-related Breast Cancer AAMR per 100,000 stratified by race in the United States from 1999 to 2020.
