# Supplementary material for: Impact of diabetes on breast cancer mortality in elderly female patients: A retrospective analysis (1999–2020)
Source: Medicine (Baltimore). 2026 May 22;105(21):e48934. doi: 10.1097/MD.0000000000048934 (PMC13200986; doi:10.1097/MD.0000000000048934)
Supplement: Supplementary file 7 [file medi-105-e48934-s007.docx]

| **Age Adjusted Rate (95% CI)** | | | | |
| --- | --- | --- | --- | --- |
| **Year** | **Northeast** | **Midwest** | **South** | **West** |
| 1999 | 10.5(9.5-11.4) | 12.3(11.3-13.3) | 8.6(8-9.3) | 8.6(7.7-9.6) |
| 2000 | 11.4(10.4-12.3) | 12.5(11.5-13.4) | 8.9(8.2-9.6) | 10.1(9.1-11.1) |
| 2001 | 10.5(9.5-11.4) | 12.4(11.4-13.3) | 9.6(8.9-10.3) | 9.5(8.6-10.5) |
| 2002 | 11.2(10.3-12.2) | 12(11-12.9) | 9.7(9-10.4) | 10.9(9.9-12) |
| 2003 | 10.3(9.4-11.2) | 12.3(11.4-13.3) | 9.6(8.9-10.3) | 9.7(8.8-10.7) |
| 2004 | 10.6(9.7-11.5) | 13.1(12.1-14.1) | 9.3(8.6-10) | 10.4(9.4-11.4) |
| 2005 | 10.4(9.5-11.4) | 12.5(11.5-13.5) | 9.2(8.5-9.9) | 9.8(8.8-10.7) |
| 2006 | 10.4(9.4-11.3) | 12.6(11.6-13.6) | 8.9(8.3-9.6) | 10.8(9.8-11.8) |
| 2007 | 10.4(9.5-11.3) | 12.6(11.6-13.5) | 9.6(9-10.3) | 9.2(8.3-10.1) |
| 2008 | 9.9(9-10.8) | 12.6(11.6-13.5) | 9.3(8.7-10) | 9(8.1-9.9) |
| 2009 | 9.3(8.4-10.2) | 12.2(11.3-13.1) | 9(8.3-9.6) | 10.3(9.4-11.2) |
| 2010 | 10(9.1-10.9) | 11.6(10.7-12.6) | 9.3(8.7-10) | 10.2(9.3-11.1) |
| 2011 | 9.1(8.3-10) | 11.3(10.4-12.2) | 9(8.3-9.6) | 9.7(8.8-10.5) |
| 2012 | 8.8(8-9.7) | 10.9(10-11.7) | 9.2(8.5-9.8) | 9.9(9-10.7) |
| 2013 | 8.2(7.4-9) | 9.9(9.1-10.7) | 9.3(8.7-9.9) | 9.8(8.9-10.6) |
| 2014 | 8.3(7.5-9.1) | 10.4(9.5-11.2) | 8.4(7.8-9) | 9.2(8.4-10) |
| 2015 | 8.5(7.7-9.3) | 10.4(9.6-11.3) | 8.2(7.6-8.8) | 8.2(7.5-8.9) |
| 2016 | 7.6(6.8-8.4) | 9.9(9.1-10.7) | 8.8(8.2-9.4) | 9.3(8.5-10.1) |
| 2017 | 7.3(6.6-8) | 8.9(8.2-9.7) | 8.3(7.8-8.9) | 9.5(8.7-10.3) |
| 2018 | 8.3(7.5-9.1) | 8.8(8.1-9.6) | 8.8(8.3-9.4) | 9.8(9-10.6) |
| 2019 | 8.1(7.3-8.8) | 9.5(8.7-10.2) | 9.6(9.1-10.2) | 9(8.2-9.7) |
| 2020 | 10.2(9.4-11) | 11.3(10.5-12.1) | 11.8(11.1-12.4) | 10.3(9.6-11.1) |

**Supplementary Table 5.** Diabetes-related Breast Cancer AAMR per 100,000 stratified by Census Region in the United States from 1999 to 2020.
