# Supplementary material for: Impact of diabetes on breast cancer mortality in elderly female patients: A retrospective analysis (1999–2020)
Source: Medicine (Baltimore). 2026 May 22;105(21):e48934. doi: 10.1097/MD.0000000000048934 (PMC13200986; doi:10.1097/MD.0000000000048934)
Supplement: Supplementary file 8 [file medi-105-e48934-s008.docx]

| **State** | **Age-Adjusted Rate (95% CI)** |
| --- | --- |
| District of Columbia | 15.8(13.3-18.3) |
| Ohio | 15.6(15.1-16.1) |
| Nebraska | 15.4(14.1-16.8) |
| Oklahoma | 14.3(13.4-15.2) |
| West Virginia | 13.7(12.6-14.9) |
| Mississippi | 12.8(11.8-13.8) |
| Minnesota | 12.7(12-13.4) |
| North Dakota | 12.7(10.8-14.6) |
| Vermont | 12.6(10.6-14.7) |
| Oregon | 12.5(11.7-13.3) |
| Wyoming | 12.5(10.1-14.9) |
| Kentucky | 12.2(11.4-13) |
| Maryland | 11.8(11.2-12.5) |
| Pennsylvania | 11.8(11.4-12.2) |
| Indiana | 11.2(10.6-11.8) |
| South Dakota | 11.2(9.5-12.8) |
| Texas | 11.2(10.8-11.6) |
| Iowa | 11.1(10.3-12) |
| North Carolina | 11(10.5-11.5) |
| California | 10.8(10.5-11.1) |
| Tennessee | 10.4(9.8-11) |
| Colorado | 10.3(9.5-11) |
| Rhode Island | 10.3(9-11.7) |
| Washington | 10.2(9.6-10.8) |
| Michigan | 9.9(9.4-10.4) |
| Wisconsin | 9.9(9.3-10.5) |
| Missouri | 9.8(9.2-10.4) |
| New Jersey | 9.7(9.2-10.2) |
| South Carolina | 9.5(8.8-10.2) |
| Idaho | 9.4(8.2-10.6) |
| Alaska | 9.3(7-12.2) |
| Maine | 9.3(8.2-10.4) |
| New Hampshire | 9.1(7.9-10.3) |
| Louisiana | 8.9(8.2-9.6) |
| Montana | 8.8(7.5-10.2) |
| Illinois | 8.7(8.3-9) |
| Kansas | 8.6(7.8-9.4) |
| Virginia | 8.6(8.1-9.1) |
| Hawaii | 8.5(7.4-9.6) |
| New York | 8.5(8.2-8.8) |
| Connecticut | 8.3(7.6-9) |
| Utah | 8.2(7.2-9.3) |
| Arkansas | 8(7.2-8.7) |
| Alabama | 7.8(7.3-8.4) |
| Georgia | 7.3(6.9-7.8) |
| Delaware | 7.2(5.9-8.4) |
| Massachusetts | 7.2(6.7-7.7) |
| New Mexico | 7(6.1-7.9) |
| Florida | 5.1(4.9-5.3) |
| Arizona | 4.8(4.4-5.2) |
| Nevada | 3.6(3-4.2) |

**Supplementary Table 6.** Diabetes-related Breast Cancer AAMR per 100,000 stratified by state in the United States from 1999 to 2020.
