# Supplementary material for: Impact of diabetes on breast cancer mortality in elderly female patients: A retrospective analysis (1999–2020)
Source: Medicine (Baltimore). 2026 May 22;105(21):e48934. doi: 10.1097/MD.0000000000048934 (PMC13200986; doi:10.1097/MD.0000000000048934)
Supplement: Supplementary file 9 [file medi-105-e48934-s009.docx]

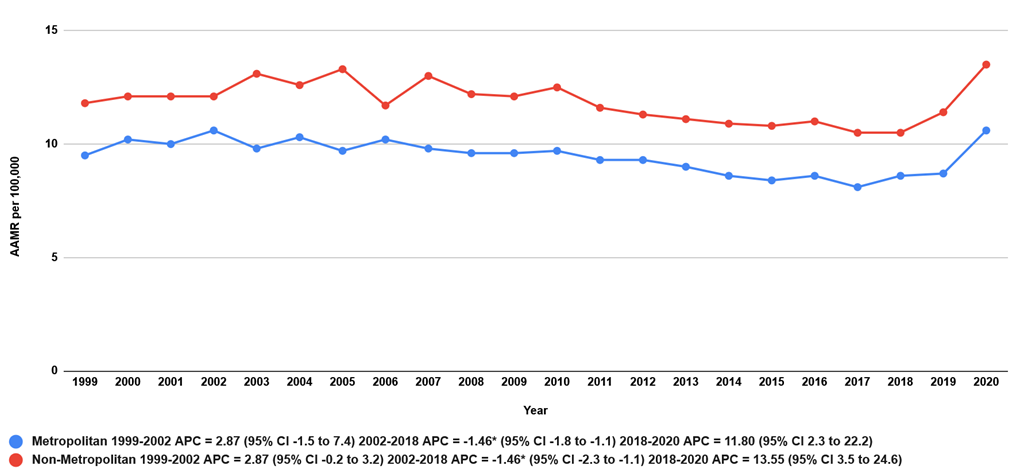


**Supplementary Figure 3.** Diabetes-related Breast Cancer AAMR per 100,000 stratified by Urban-Rural classification in the United States from 1999 to 2020.
