# Supplementary material for: Impact of diabetes on breast cancer mortality in elderly female patients: A retrospective analysis (1999–2020)
Source: Medicine (Baltimore). 2026 May 22;105(21):e48934. doi: 10.1097/MD.0000000000048934 (PMC13200986; doi:10.1097/MD.0000000000048934)
Supplement: Supplementary file 10 [file medi-105-e48934-s010.docx]

| **Age-Adjusted Rate (95% CI)** | | |
| --- | --- | --- |
| **Year** | **Metro** | **Non-Metro** |
| **1999** | 9.5(9-9.9) | 11.8(10.7-12.8) |
| **2000** | 10.2(9.7-10.6) | 12.1(11-13.2) |
| **2001** | 10(9.5-10.5) | 12.1(11.1-13.2) |
| **2002** | 10.6(10.1-11) | 12.1(11.1-13.2) |
| **2003** | 9.8(9.4-10.3) | 13.1(12-14.2) |
| **2004** | 10.3(9.8-10.7) | 12.6(11.5-13.7) |
| **2005** | 9.7(9.3-10.2) | 13.3(12.1-14.4) |
| **2006** | 10.2(9.7-10.7) | 11.7(10.7-12.8) |
| **2007** | 9.8(9.4-10.3) | 13(11.9-14.1) |
| **2008** | 9.6(9.2-10.1) | 12.2(11.2-13.3) |
| **2009** | 9.6(9.2-10.1) | 12.1(11-13.1) |
| **2010** | 9.7(9.2-10.1) | 12.5(11.4-13.6) |
| **2011** | 9.3(8.8-9.7) | 11.6(10.5-12.6) |
| **2012** | 9.3(8.9-9.7) | 11.3(10.3-12.3) |
| **2013** | 9(8.5-9.4) | 11.1(10.1-12.1) |
| **2014** | 8.6(8.2-9) | 10.9(9.9-11.9) |
| **2015** | 8.4(8-8.7) | 10.8(9.8-11.7) |
| **2016** | 8.6(8.2-8.9) | 11(10.1-12) |
| **2017** | 8.1(7.8-8.5) | 10.5(9.6-11.5) |
| **2018** | 8.6(8.3-9) | 10.5(9.6-11.4) |
| **2019** | 8.7(8.4-9.1) | 11.4(10.4-12.3) |
| **2020** | 10.6(10.2-11) | 13.5(12.5-14.6) |

**Supplementary Table 7.** Diabetes-related Breast Cancer AAMR per 100,000 stratified by Urban-Rural classification in the United States from 1999 to 2020.
